# Identification of a novel homozygous *LAMB3* mutation in a Chinese male with junctional epidermolysis bullosa and severe urethra stenosis: A case report

**DOI:** 10.3389/fgene.2022.965375

**Published:** 2022-09-30

**Authors:** Wei Wang, Qiang Guo, Jinshan Chen, Xi Zhang, Chengyong Li, Shuangping Li, Jialin Liang, Chuan Hao, Jingqi Wang

**Affiliations:** ^1^ Department of Urology, The Second Hospital of Shanxi Medical University, Taiyuan, China; ^2^ The Second Medicine College, Shanxi Medical University, Taiyuan, China

**Keywords:** epidermolysis bullosa, LAMB3, mutation, urological complications, urethral stricture

## Abstract

**Introduction:** Epidermolysis bullosa (EB) is a skin fragility disorder that is caused by molecular aberrations in the epidermal basement membrane zone. Based primarily on the cleavage plane within the skin, EB is classified into four major subtypes: EB simplex; junctional EB (JEB); dystrophic EB; and Kindler EB. The junctional form (JEB) can lead to blistering and a variety of extracutaneous complications, including genitourinary tract involvement. Despite therapeutic progress, treatment modalities for urological complications of JEB are currently limited.

**Results:** We present the case of a Chinese male with intermediate JEB and profound urinary tract stenosis. Due to the progression of the urinary tract stenosis, he presented with repeated urological symptoms, such as high frequency of urination, painful urination, and difficult voiding. After birth, multiple blisters on the fingers, feet, and limbs, as well as nail dystrophies and spare hair were noted. Mutation analysis revealed that the patient carried a homozygous frameshift mutation in the *LAMB3* gene [c.1172_1179delinsTGTGTGTGCAAGGAG/p. (P391Lfs*23)]. After receiving treatment for urethral dilatation, lingual mucosa for anterior urethroplasty, and repair of urethral stricture using a ventral onlay penile skin flap, the patient still experienced a relapse of urinary tract stenosis. Finally, the patient underwent perineal urethrostomy. In contrast, his older brother with similar urological symptoms received regular urethral dilatation, and the curative effect was positive.

**Conclusion:** Here we report on a case with a novel *LAMB3* mutation that led to JEB with profound urinary tract stenosis, which has expanded our experience in the treatment of EB urological complications.

## 1 Introduction

Inherited epidermolysis bullosa (EB) is a group of rare genetic dermatoses characterized by mucocutaneous fragility and blisters after minimal trauma or friction. As a severe form of the condition, junctional EB (JEB) is associated with mutations in seven genes encoding for structural proteins in the epidermal basement membrane zone, comprising type XVII collagen (*COL17A1*), laminin-332 (*LAMA3*, *LAMB3* and *LAMC2*), integrin α6β4 (*ITGA6* and *ITGB4*), and integrin α3 subunits (*IGTA3*) (Bardhan et al., 2020).

Among the various structural proteins, laminin-332 (LM-332) is an integral component of the epidermal basement membrane and is encoded by *LAMA3*, *LAMB3*, and *LAMC2*. It is highly abundant in the skin. Patients with *LAMB3* mutations present with various phenotypes, including severe or intermediate JEB and amelogenesis imperfecta (McGarth et al., 1996; Yuen et al., 2011; Kiritsi et al., 2015; Smith et al., 2019). Due to the presence of LM-332 in multiple epithelial basement membranes outside the skin, JEB with *LAMB3* mutations can cause severe extracutaneous complications. Complications in epithelial-associated tissues can be found within several organ systems, including the gastrointestinal and genitourinary tracts (Fine and Mellerio, 2009a). However, complications can be found in many other organs, such as the heart, kidney, and teeth (Fine and Mellerio, 2009b). Notably, a small proportion of patients with JEB may present with severe urological complications, which can greatly affect their quality of life (Fine et al., 2004; Chan et al., 2007). However, experience in the treatment of EB urological complications is still lacking. Meatotomy, subureteric injections of a bulking agent, and ureteric stenting are helpful. Furthermore, medical therapy may help relieve the voiding dysfunction (Kajbafzadeh et al., 2010). Other common treatments include ureterosigmoidostomy, bladder reconstruction, the fashioning of a Mitrofanoff channel, and urinary diversion (Donatucci et al., 1992; Glazier and Zaontz, 1998; Chan et al., 2007).

Here, we present the case of a Chinese male with JEB with profound urethral stricture due to a novel homozygous *LAMB*3 mutation. To deepen our understanding of this disease, we explore the surgical treatment of urinary complications associated with EB.

## 2 Methods

### 2.1 Whole-exome sequencing analysis

Genomic DNA was extracted from patients’ peripheral blood samples for library preparation. After fragmentation, joint connection, amplification, and purification, libraries were constructed using hybrid capture. The target region, including the coding sequence of the 20,099 genes in human whole exomes and their neighboring 20-base pair intron regions, was detected using a high-throughput sequencing platform. The variants were acquired and annotated by comparing the sequencing data to the GRCh37/hG19 reference sequence of the human genome. After that, the variants were filtered ulteriorly according to the proband’s clinical phenotype (EB). A total of 31 genes (Supplemental Table S1) that were associated with EB were included in the filtration list. After the filtration, the variants’ pathogenicity was evaluated according to the American College of Medical Genetics and Genomics (ACMG) guidelines. Ultimately, the suspected pathogenic variants were verified in the proband, his brother, and parents using Sanger sequencing.

## 3 Case description

### 3.1 Presenting concerns

A 29-year-old male (height, 1.82 m; weight, 60 kg), the second child of healthy non-consanguineous Chinese parents, presented to our clinic with a complaint of difficult voiding, painful urination, and weak urinary stream. The patient’s older brother showed similar clinical features, including difficult voiding and generalized skin lesions.

At the age of 27 years, due to difficult voiding with frequent and painful urination, the patient presented to the local hospital and was diagnosed with urethral infection. Anti-infective therapy was started, and the patient’s symptoms were partially relieved. During the next 2 years, frequent, urgent, and/or painful urination occurred discontinuously with a tapering urinary stream. Suffering from progressive difficult voiding with occasional hematuria, the patient took oral anti-infective drugs, and this had minimal effect. He was admitted to the Second Hospital of Shanxi Medical University for further diagnosis and treatment. Physical examination showed that the skin of his penis and scrotum was normal, and there were no abnormalities in the opening of the external orifice of the urethra. In addition, he had mechanically induced blistering on his left knee since birth, and sparse blistering on his fingers, feet, and lower extremities. The blisters healed with scarring, post-inflammatory hyperpigmentation, and atrophy. Nail dystrophies developed in early infancy, leading to the loss of all fingernails and toenails in childhood. His hair was sparse and presented with progressive loss of body and axillary hair in adulthood. In addition, enamel hypoplasia and pitting were also observed. However, no pyloric atresia or signs of digestive tract obstruction were observed. Physical examination showed severe atrophy of the distal joint, exfoliation of a large area of skin, and ulcerations on a part of the skin. The patient was diagnosed as suffering from JEB at the age of 8 years, and as being a hepatitis B virus carrier at the age of 10 years. Furthermore, at the age of 12 years, the patient was diagnosed with acute nephritis, which was thought to be a JEB complication, and was cured. The patient’s serum creatinine remained within the normal range during his stay in our department.

### 3.2 Clinical findings

**FIGURE 1:**
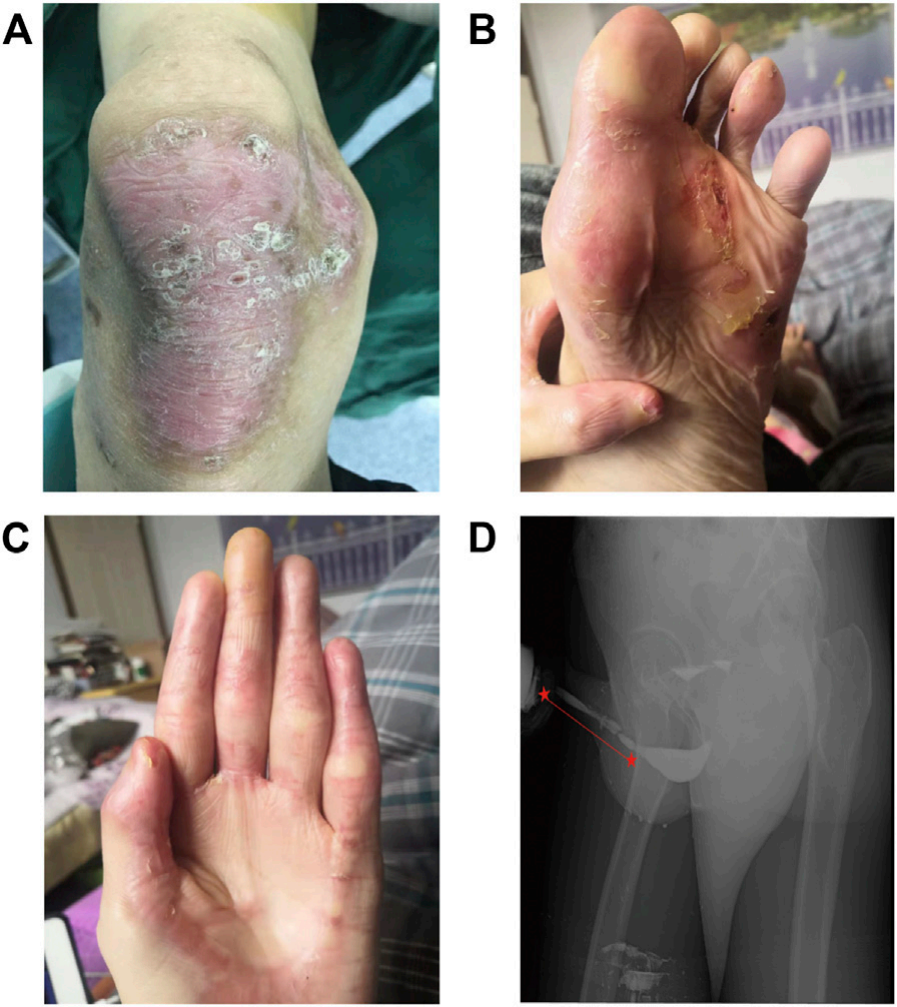
**(A–C)** Blisters and scarring on the knees, feet, and fingers, respectively. **(D)** Retrograde urethrography showed stricture at the cavernous part of urethra and inflated bulbar urethra.

Various degrees of scarring and hyperpigmentation were found on the corners of the patient’s mouth, as well as on his fingers, feet, and extremities, especially the knees (Figures 1A–C). The patient was a male youth who had no medical history of trauma or medical operation. However, over the past 2 years, he had experienced a tapered urinary stream and progressive difficult voiding, which was thought to be a symptom of urethral stricture caused by JEB. To obtain a definite diagnosis, various examinations were performed.

### 3.3 Diagnostic focus and assessment

#### 3.3.1 Imaging examination

Retrograde urethrography was performed. Iohexol was injected into the bladder through the external urethral orifice and observation and filming were performed simultaneously. Stricture at the cavernous urethra (∼8.5 cm long) and an inflated bulbar urethra were found (Figure 1D). Furthermore, color ultrasound showed urinary retention and a slightly larger than normal prostate with a size of about 4.9*3.5*3.4 cm.

#### 3.3.2 Mutation identification

**FIGURE 2:**
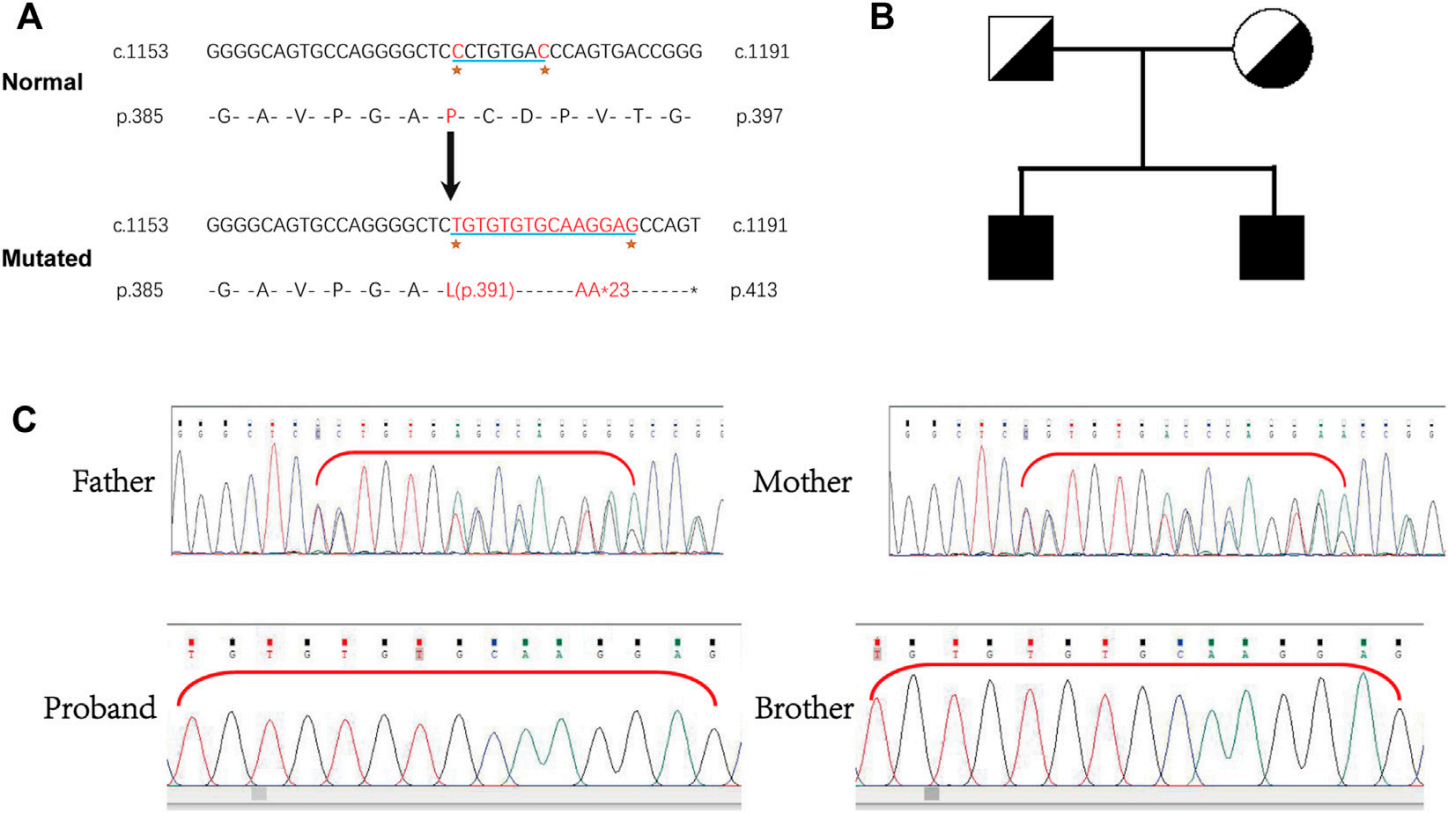
**(A)** Exome sequencing identified a homozygous mutation, *LAMB3*: NM_000228.2: exon11: c.1172_1179delinsTGTGTGTGCAAGGAG: p.P391Lfs*23 in the patient. **(B)** Pedigree showing unaffected parents and two affected brothers. **(C)** Homozygosity of this mutation in the patient and his older brother was confirmed by Sanger sequencing, and the clinically unaffected parents were heterozygous carriers.

Whole-exome sequencing was performed, and a novel homozygous *LAMB3* mutation was found. Sanger sequencing confirmed that the novel mutation (chr1:209801489-209801496, NM_000228.2, c.1172_1179delinsTGTGTGTGCAAGGAG) in exon 11 of *LAMB3* was homozygous in the patient and his older brother, with a predicted coding sequence change of p.P391Lfs*23. Furthermore, the clinically unaffected parents were heterozygous carriers of the corresponding mutation (Figure 2C), indicating a recessive genetic disease (Figure 2B). Bases 1172-1179 of *LAMB3* were lost and TGTGTGTGCAAGGAG was inserted in this position, which led to a transition from proline to leucine on the 391st amino acid, resulting in a frameshift and truncated protein (Figure 2A). This mutation is a novel variant of *LAMB3*-associated JEB that has not been reported in the OMIM and gnomAD databases.

### 3.4 Timeline

**Table:** 

Dates	Relevant past medical history and interventions		
1998–2019	JEB was diagnosed in 1998. From 2017 to 2019, the patient suffered from difficult voiding with frequent and painful urination and received anti-infective treatment. In his family, his parents did not have any symptoms similar to him. However, his older brother also had difficult voiding.		
Date	Summaries from initial and follow-up visits.	Diagnostic testing (including dates)	Interventions
2019-02-14–2019-02-25	Primary concerns: difficult voiding with frequent and painful urination for 2 years.	Retrograde urethrography (2019-02-15)	Lingual mucosal urethroplasty (2019-02-25)
	Diagnose: cavernous urethral stricture and JEB.		
2019-05–2019-11	Five weeks after catheter removal, the patient had a weak urinary stream again.		Regular urethral dilatation
2019-12–2021-02	After the COVID-19 pandemic, the patient stopped receiving urethral dilatation and experienced progressive, difficult voiding.		Penile skin flap urethroplasty (2021-02-18)
2021-02–2021-05	The patient experienced trickle micturition 6 weeks after the penile skin flap urethroplasty. Retrograde urethrography showed that his urethra was close to atresia.		Perineal urethrostomy (2021-05-26)
2021-05-26 to present	The patient had normal voiding after the urethrostomy.		

### 3.5 Therapeutic focus and assessment

**FIGURE 3:**
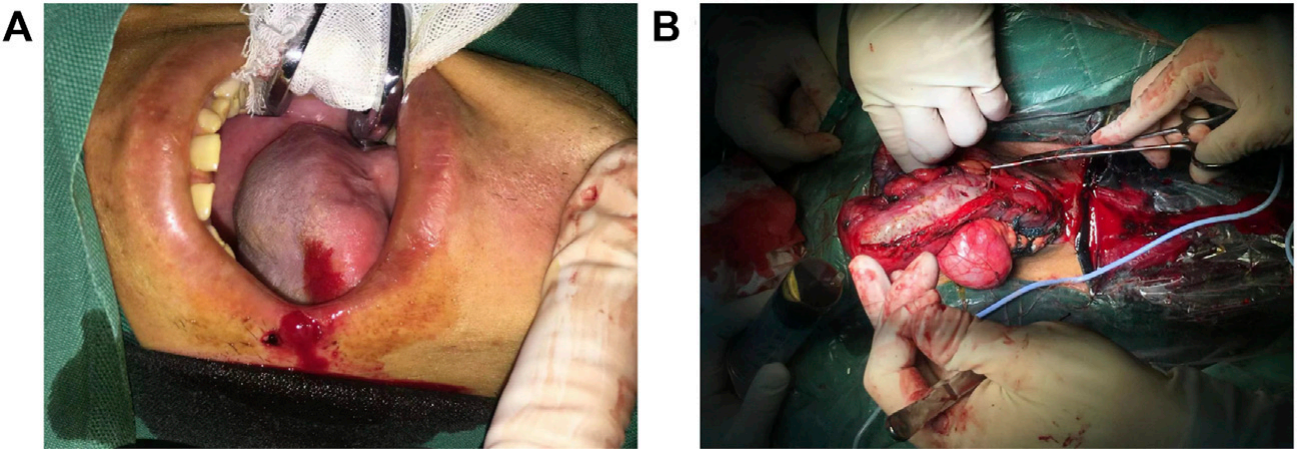
**(A)** Extraction of tongue mucosa. **(B)** The mucosa graft was sutured to the urinary tract.

The patient was diagnosed with cavernous urethral stricture, and anti-infective therapy, oral cleaning, and cleaning of the penis, scrotum, perineum, and anus were performed before the operation. Lingual mucosal urethroplasty was performed under general anesthesia (Figures 3A,B). Postoperatively, the pathological results of the urethral mass showed squamous metaplasia with keratosis. Submucosal inflammatory granulation tissue hyperplasia and focal adenoid structures of the small nests were noted, but no cell atypia was observed. The catheter was removed 4 weeks after the operation, and the patient voided with a good stream. Five weeks after catheter removal, the patient had a weaker urinary stream than before and started to undergo urethral dilatation once or twice weekly in the outpatient clinic, which lasted for half a year. Due to the COVID-19 pandemic, the patient could not continue with urethral dilatation. As a result, he experienced progressive, difficult voiding, and trickle micturition. On February 18, 2021, the patient underwent penile skin flap urethroplasty at the Sixth People’s Hospital affiliated with Shanghai Jiaotong University. At this time, the patient could not void normally after catheter removal and experienced trickle micturition 6 weeks after the operation. Retrograde urethrography could not be performed successfully in the patient because the contrast medium could not be injected into the bladder. This indicated that the patient’s urethra was close to atresia. Finally, the patient underwent perineal urethrostomy under general anesthesia on May 26, 2021. He had normal voiding after the urethrostomy. In contrast, his older brother underwent urethral dilatation using urethral sound 18 in an outpatient setting once or twice bi-weekly. As a result, he has maintained the patency of urination.

## 4 Discussion

As a group of rare genetic dermatoses, EB affects quality of life to varying degrees and can even lead to mortality. More than 30 subtypes of EB have been recognized thus far, most of which are associated not only with classical symptoms of mucocutaneous fragility and blister formation but also severe extracutaneous manifestations (Bardhan et al., 2020). Infrequent cases and the heterogeneity of the disorder have significantly hindered our deep exploration of the disease. Therefore, assessing clinical problems, disease complications, and optimal treatments for various subtypes of EB are all necessary to develop effective treatment, which is urgently needed (Davila-Seijo et al., 2014).

Depending on the plane of cleavage within the skin, EB disorders are classified into four major categories: JEB (intralamina lucida); EB simplex (intraepidermal); dystrophic EB (DEB; sublamina densa); and Kindler EB (variable and mixed). Recognized as a relatively rare but vital subtype of EB, JEB is inherited in an autosomal recessive pattern and is characterized by decreased dermal-epidermal adhesion due to the loss or lack of normal LM-332, XVI collagen, integrin α6β4, or integrin α3 (Condrat et al., 2018). JEB is divided into two phenotypes according to the degree of lethality, namely severe and intermediate JEB (Has et al., 2020). Similar to severe JEB, intermediate JEB is characterized by generalized blisters. However, in contrast to other EB subtypes, atrophic scars, hypopigmentation, or hyperpigmentation at the sites of healed blisters have been noted in adults with intermediate JEB (Yancey and Hintner, 2010).

As an important member of the laminin family, LM-332 is comprised of α3β3γ2, which is encoded by *LAMA3*, *LAMB3*, and *LAMC2* (Domogatskaya et al., 2012). As a major component of anchoring filaments in the skin, LM-332 can simultaneously bind to cellular receptors (such as integrin α6β4 and α3β1) and other matrix proteins, forming critical anchoring junctions and/or networks for epithelial cell adhesion and wound healing. Therefore, the loss of functional LM-332 may cause JEB. The mechanism involves frictional forces that separate the whole epidermis from the basement membrane both on the skin and mucosal surfaces, exposing these regions to pathogens, causing fluid loss, and compromising the skin’s regulatory functions (Has et al., 2018).

Mutations in *LAMA3*, *LAMB3*, and *LAMC2* can all result in JEB (Has et al., 2018). With the highest mutation frequency, *LAMB3* has a complex mutation spectrum comprising nonsense, missense, splice site, small insertions/deletions, gross deletions and insertions, and unconventional intronic mutations (Hammersen et al., 2016). In 1994, the first mutation in the *LAMB3* gene causing severe JEB was detected (Pulkkinen et al., 1994). c.1903C>T/p. (R635X), the most common *LAMB3* pathogenic variant, is the most common cause of severe JEB. Most patients with EB with *LAMB3* mutations are reported to have generalized skin lesions, mucosal blistering, and generalized or focal enamel hypoplasia (Sadler et al., 2005), and a few patients also present with persistent ocular involvement with unremitting and painful corneal abrasions (Castiglia et al., 2021). More seriously, many patients with a severe JEB phenotype caused by *LAMB3* mutations have been reported to die within a year after birth. Given the heterogeneity of JEB, we systematically reviewed the *LAMB3* mutation spectrum of JEB (Table 1). A total of 93 *LAMB3* mutations leading to JEB were found (Supplemental Table S2). However, patients with JEB associated with urological complications have seldom been reported to harbor mutations in *LAMB3* until now.

**Table:** 

JEB caused by LAMB3 mutations in the past (Partial)					
Author	Age	Sex	Subtypes	Mutation sites	Urological symptoms
Hata, D. et al.2005	4 months	M	H-JEB	c.2379de1G/c.2938C>T	massive albuminuria
Yenamandra, V. K. et al.2017	N	M	nH-JEB	c.1063T>C/c.1063T>C	difficulty in micturition and urethral stenosis
Suci Wdhiati et al.2021	10 years;22years	M;F	JEB-GI	c.962A>C/c.962A>C	N
Kourosh Riahi, M.D. et al.2021	7 years	F	JEB	c.1405T>C	N
Fehmida F. Khan et al.2021	N	M	H-JEB	c.1705C>T/c.1705C>T	N
Hung, J. H. et aI.2021	26 year	F	JEB	c.373-9T>A/c.3119G>A	N
				c.972delA/c.972de1A	
				c.1978C>T/c.1978C>	
Raghad Alharthi et al.2021	N	N	JEB	c.958_1034dup/c.958_1034dup	N
				c.1977-1 G>A/c.1977-1 G>A	
				c.3052-	
Daniele Castiglia et aI.2021	6 years	F	JEB	N 5C>G/c.3492_3493deICG	N

The uniqueness of our patient lies in the fact that genetic testing revealed a homozygous insertion/deletion mutation in *LAMB3*. In *LAMB3*, the 10th coding area of exon 11 had a deletion-insertion mutation (c.1172_1179delinsTGTGTGTGCAAGGAG) leading to a frameshift and resulting in truncation of the produced protein (p.P391Lfs*23). This is a novel mutation that has not been previously reported in population databases, such as gnomAD (https://gnomad.broadinstitute.org/). Based on the ACMG criteria and our patient’s profile, we believe that it is pathogenic. The *LAMB3* gene is associated with intermediate JEB, which is similar to our patient’s phenotype. On the other hand, the patient’s homozygosity of the mutation, without either parent presenting with a cutaneous phenotype, implies that the mutations have an autosomal recessive inheritance pattern. More importantly, in addition to blisters and ulcers on the skin, our patient also experienced significant urinary complications.

Because any epithelial-lined organ can be damaged by EB, it is recognized that EB can have multisystem involvement. Regarding extracutaneous manifestations of EB, some EB subtypes are at risk of squamous cell carcinoma (SCC), basal cell carcinoma, or malignant melanoma, as well as serious harm to the bone marrow, eye, gastrointestinal tract, musculoskeletal system, heart, kidney, and teeth (Fine and Mellerio, 2009b). For example, JEB has been known to cause interstitial lung disease and nephrotic syndrome. Furthermore, some types of EB, particularly severe recessive dystrophic EB, are associated with a much higher rate of aggressive skin and mucosal SCC (Robertson et al., 2021).

Urological complications have been reported in a few patients. An epidemiological study in America showed that urinary tract complications occurred most frequently in patients with severe JEB, among which urethral meatus stenosis was the most common complication (Fine et al., 2004). Severe or mild JEB and DEB, as well as Kindler EB, can cause genitourinary tract involvement.

The most common urological manifestation of EB is severe hematuria, although patients may also present with meatal stenosis, sepsis, dysuria, ureterovesical junction blockage, and hydronephrosis (Valari et al., 2019). In addition, scarring of the glans penis/labia has also been reported to occur at a relatively high frequency (Kajbafzadeh et al., 2010). In some cases of EB, genitourinary tract involvement can be substantial, leading to bladder distension, hydroureter, and hydronephrosis, eventually leading to chronic renal failure if left untreated (Almaani and Mellerio, 2010). Patients with EB have also been reported to develop blistering of the bladder mucosa and thickening and fibrosis of the bladder wall (Eklöf and Parkkulainen, 1984). Renal parenchymal disease has three main forms (IgA nephropathy, post-infectious glomerulonephritis, and secondary renal amyloidosis), which can all lead to renal failure, even if treatable problems are addressed (Tammaro et al., 2008). Concerning genitourinary tract monitoring in patients with EB, they may benefit from annual ultrasound imaging. To identify obstructive lesions and assess contemporaneous vesico-ureteric reflux (VUR), voiding cystourethrography and diuretic renography should be performed. If any abnormalities are found, imaging or functional testing should be performed (Fine et al., 2004). As a key component of the hemidesmosome-anchoring complex, integrin α6β4 is encoded by *ITGA6* and *ITGB4*. In the past, JEB with pyloric atresia (JEB-PA) caused by integrin α6β4 mutations was universally thought to be associated with early onset pyloric atresia. However, for some non-lethal variants, it is not always a hallmark of integrin-associated JEB, and urological issues are common (Yoshida et al., 2019). Unspecific cystitis, dysuria, recurrent vesicourethral occlusion, bladder wall changes, hydronephrosis, pyelonephritis, and nephroliths have been found in patients with JEB-PA (Lee et al., 2015).

Considering the interaction of LM-332 with integrin α6β4, we speculated that mutations in genes encoding structural domains linked to integrin α6β4 may cause urological symptoms similar to those of mildly symptomatic patients with mutations in *ITGA6* or *ITGB4*. The low number of previously reported cases of JEB with *LAMB3* mutations may be due to the fact that only a few of the previous cases had mutations in *LAMB3* or that they had a short survival period.

Thus far, a concerted effort has been made to identify treatment options for EB complications. These studies have made many important findings that have improved the prognosis of patients with EB. For instance, a two-step multidisciplinary approach may improve malnutrition and growth retardation in recessive dystrophic epidermolysis bullosa (RDEB) patients (Vowinkel et al., 2015).

Regarding treatment for EB urological complications, operative therapy has been proven effective. Meatotomy has been shown to be helpful in treating urological symptoms in patients with JEB, including urinary retention and voiding difficulty. Four patients (2 with JEB, 2 with DEB) with VUR received a urocol bulking agent injected into their ureters, and a ureteric stent was implanted to widen the stenosis caused by the blistering sores. Consequently, their symptoms were resolved, and they had no ulcers or scars (Kajbafzadeh et al., 2010). Two brothers with JEB were reported to have recalcitrant dysuria and urinary tract obstruction caused by meatal stenosis, which was temporarily relieved by meatotomy but recurred repeatedly after the operation. Finally, the brothers underwent ureterosigmoidostomy. Although it caused postoperative complications such as metabolic acidosis, the operation significantly relieved urological symptoms (Glazier and Zaontz, 1998). For patients with DEB and symptomatic phimosis, classical circumcision may be more appropriate than applying a Plastibell clamp with a touchless technique (Jesus et al., 2014). It is worth noting that male patients with EB who did not receive circumcision have been found to be likely to present with severe painful phimosis during adulthood, resulting in acute urinary retention (Fine et al., 2004). On the other hand, drug therapy has also been found to be helpful in some situations as α-blockers are associated with improved detrusor instability and bladder compliance in patients with JEB or DEB (Kajbafzadeh et al., 2010).

In EB, recurrent blisters and erosion in the urethra may lead to scarring and, as a result, blockage of the urethral meatus. As a surgical method currently used to treat urethral meatus on a large scale in the clinic, lingual mucosa for anterior urethroplasty consists of extraction of the lingual mucosa, exposure of the urethral lumen, and suturing the lingual mucosa graft to the urinary tract (Horiguchi, 2017). After undergoing lingual mucosa for anterior urethroplasty and urethroplasty using a ventral onlay penile skin flap, the patient in this study still developed intractable dysuria. This may indicate that such invasive operations may lead to progressive scarring and may not have a significant therapeutic effect. In contrast, the patient’s older brother underwent regular urethral dilatation and did not suffer significant urethral stenosis. Two different treatments and matched prognoses for the two brothers has provided us with valuable experience in the treatment of urinary tract complications in patients with EB. Furthermore, the treatment experience of the older brother indicated that timely urethral dilatation when finding a urethral stricture may be a way to treat this urological complication.

The present study identified a naturally occurring *LAMB3* mutation in an adult patient with JEB who presented with profound urethral stricture and recurrent difficult voiding. With the same *LAMB3* homozygous mutation, the patient’s older brother presented with much milder urethral stricture. The difference in therapy between the two brothers may explain the different clinical manifestations. As a novel *LAMB3* homozygous mutation was identified in our patient, our current knowledge of the *LAMB3* mutation spectrum has been expanded.

## Data Availability

The datasets for this article are not publicly available due to concerns regarding participant/patient anonymity. Requests to access the datasets should be directed to the corresponding author.
