# Supplementary material for: Identification of a novel homozygous LAMB3 mutation in a Chinese male with junctional epidermolysis bullosa and severe urethra stenosis: A case report
Source: Front Genet. 2022 Sep 30;13:965375. doi: 10.3389/fgene.2022.965375 (PMC9561087; doi:10.3389/fgene.2022.965375)
Supplement: Supplementary file 2 [file Table2.DOCX]

| **JEB caused by *LAMB3* mutations in the past** | | | | | | |  |
| --- | --- | --- | --- | --- | --- | --- | --- |
| **Authors/years** | **Age** | **Sex** | **Genes** | **Subtypes** | **Mutation sites** | **Urological symptoms** |  |
| Hata, D. et al.2005 | **4 months** | M | LAMB3 | H-JEB | c.2379delG/c.2938C>T | massive albuminuria |  |
| Yenamandra, V. K. et al.2017 | **3 months** | M | LAMB3 | H-JEB | c.1705C>T/c.1705C>T | N |  |
|  | N | M |  | nH-JEB | c.1063T>C/c.1063T>C | N |  |
|  | N | M |  |  |  | difficulty in micturition and urethral stenosis |  |
|  | N | F |  |  |  | N |  |
| Suci Widhiati et al.2021 | 10 years | M | LAMB3 | JEB-GI | c.962A>C/c.962A>C (p.H321P) | N |  |
|  | 22years | F |  |  |  |  |  |
| Kourosh Riahi, M.D. et al.2021 | 7 years | F | LAMB3 | JEB | c.1405T>C (p.Y339H) | N |  |
| Fehmida F . Khan et al.2021 | N | M | LAMB3 | H-JEB | c.1705C>T/c.1705C>T (p.(Arg569*)) | N |  |
| Hung, J. H. et al.2021 | 26 years | F | LAMB3 | JEB | c.373-9T>A/c.3119G>A | N |  |
| Raghad Alharthi et al.2021 | N | N | LAMB3 | JEB | c.972delA/c.972delA | N |  |
|  |  |  |  |  | c.1978C>T/c.1978C>T |  |  |
|  |  |  |  |  | c.958_1034dup/c.958_1034dup |  |  |
|  |  |  |  |  | c.1977-1G>A/c.1977-1G>A |  |  |
| Daniele Castiglia et al.2021 | 6 years | F | LAMB3 | JEB | c.3052-5C>G/c.3492_3493delCG | N |  |
| Paola Fortugno et al.2020 | 61 years | M | LAMB3 | JEB-GI | c.1903C>T/c.3513C>A | N |  |
| R. Mittwollen et al.2020 | 23 years | M | LAMB3 | JEB | c.628G>A/c.1903C>T | N |  |
|  | 20 years | F |  |  |  |  |  |
| Luiza Monteavaro Mariath et al.2019 | N | N | LAMB3 | JEB | c.1132+5G>A/c.2106delG | N |  |
| Condorelli, A.G. et al.2018 | 30 years | M | LAMB3 | JEB | c.1132+5G>A/c.1132+5G>A | N |  |
| Hairong Wang, MS et al.2018 | <8 weeks | M | LAMB3 | JEB | c.822+1G>A/c.124C>T | N |  |
| Nadia Laroussi et al.2017 | N | N | LAMB3 | N | c.2701+1G>A/c.2701+1G>A | N |  |
| Fuentes, I. et al.2017 | **7 months** | M | LAMB3 | JEB | c.823-1G>A/c.957ins77 | N |  |
|  | **3 months** | F |  |  |  |  |  |
|  | **12 or 13 years** | M |  |  | c.3228+1G>A/c.3228+1G>A |  |  |
|  | 3 years | M |  |  |  |  |  |
|  | 5 years | M |  |  |  |  |  |
|  | 1 year | M |  |  |  |  |  |
|  | 7 years | M |  |  |  |  |  |
|  | **28 years** | F |  |  |  |  |  |
|  | 5 years | M |  |  |  |  |  |
|  | 1 year | F |  |  |  |  |  |
|  | 9 years | M |  |  |  |  |  |
|  | 19 years | F |  |  | c.3228+1G>A/c.3268del5 |  |  |
|  | 1 year | F |  |  | c.3228+1G>A/c.823-1G>A |  |  |
|  | **7 years** | F |  |  |  |  |  |
|  | **1 year** | F |  |  |  |  |  |
|  | 5 years | F |  |  |  |  |  |
|  | **1 year** | F |  |  |  |  |  |
|  | 12 years | F |  |  | c.3228+1G>A/c.957ins77 |  |  |
| B. Mayer et al.2016 | **4 weeks** | M | LAMB3 | H-JEB | c.1133-22G>A/c.1133-22G>A | N |  |
|  | **3 weeks** | M |  |  |  |  |  |
|  | N | N |  |  |  |  |  |
|  | N | N |  |  | c.1133-22G>A/c.1903C>T |  |  |
|  | N | N |  |  |  |  |  |
| Kiritsi, D. et al.2015 | 38 years | M | LAMB3 | JEB | c.1903C>T/c.3052-1G>A | N |  |
|  | 16 years | F |  |  | c.1903C>T/c.1288+1G>T |  |  |
| Kittridge, A. et al.2014 | **34 days** | M | LAMB3 | H-JEB | c.2842delG/c.1597G>A | N |  |
| Farooq, M. et al.2013 | 28 years | F | LAMB3 | nH-JEB | c.1460_1461insA/c.1460_1461insA | N |  |
| W.Y. Yuen et al.2011 | **<6 months** | M | LAMB3 | H-JEB | c.29-2A>G/c.957ins77 | N |  |
|  | **<1 month** | F |  |  | c.1289-2_1296del10/c.1289-2_1296del10 |  |  |
|  | **15 months** | F |  |  | c.1903C>T/c.3228+1G>T |  |  |
| M. Castori et al.2008 | **<1 year** | M | LAMB3 | H-JEB | c.31insC/c.31insC | N |  |
|  |  | M |  |  | c.241CfiT/c.241CfiT (p.R81X) |  |  |
| Castori, M. et al.2008 | **10 months** | F | LAMB3 | H-JEB | c.1945dupG/c.1945dupG | N |  |
| Anna M.G. Pasmooij et al.2007 | 46 years | M | LAMB3 | nH-JEB | c.628G>A/c.1903C>T | N |  |
|  | 64 years | M |  |  | c.628G>A/c.628G>A |  |  |
| Liu, L. et al.2006 | **5 months** | N | LAMB3 | H-JEB | c.3422delGC/c.3422delGC | N |  |
| Mühle, C. et al.2005 | **3 months** | M | LAMB3 | H-JEB | c.1903C>T/c.1628insG | N |  |
| Posteraro, P. et al.2004 | 2 months | M | LAMB3 | H-JEB | c.429G>A/c.429G>A (p.W143X) | N |  |
|  | 2 months | F |  |  |  |  |  |
|  | N | N |  |  | c.1587delAG/c.1587delAG |  |  |
| Birgit Buchroithner et al.2004 | 40 years | F | LAMB3 | JEB | c.1903C>T/c.3009C>T | N |  |
| Cserhalmi-Friedman, P. B. et al.2002 | N | N | LAMB3 | H-JEB | c.1903C>T/c.1094delA | N |  |
| Nakano, A. et al.2002 | N | N | LAMB3 | nH-JEB | c.1942delG/c.1942delG | N |  |
|  |  |  |  |  | p.Q1083X/c.1296insA |  |  |
| Hauschild, R. et al.2001 | **117 days** | M | LAMB3 | H-JEB | c.1903C>T/c.1629insG | N |  |
| Y . Gache et al.2001 | 7 years | F | LAMB3 | JEB | c.1587delAG/c.1903C>T | N |  |
|  |  |  |  |  |  |  |  |
| Cserhalmi-Friedman, P. B. et al.2001 | N | N | LAMB3 | H-JEB | c.1094delA/c.1903C>T | N |  |
| Nakano, A. et al.2000 | **<1 year** | N | LAMB3 | H-JEB | c.1903C>T/c.978delC | N |  |
|  |  |  |  |  | c.617delA/c.617delA |  |  |
|  |  |  |  |  | c.3228+2T>A |  |  |
|  |  |  |  |  | c.957ins77/c.564+5G>T |  |  |
|  |  |  |  |  | c.1101delC/c.957ins77 |  |  |
|  |  |  |  |  | c.1365delCA/c.464insT |  |  |
|  |  |  |  |  | c.1903C>T/ c.1629insG |  |  |
|  |  |  |  |  |  |  |  |
| Takizawa, Y. et al.2000 | 49 years | M | LAMB3 | GABEB | c.1977-2A>G/c.2702-29del94 | N |  |
| Cserhalmi-Friedman, P. B. et al.2000 | N | N | LAMB3 | H-JEB | c.1903C>T/c.3600A>G | N |  |
|  | N | N |  |  | c.84G>A | N |  |
| Michaela Floeth et al.1999 | 2 years | M | LAMB3 | JEB | c.1903C>T | N |  |
| John A. McGrath et al.1999 | 32 and 39 years | M | LAMB3 | GABEB | c.29insC/p.Q834X | N |  |
| L Pulkkinen et al.1998 | N | N | LAMB3 | GABEB | p.C293S/c.1367delAC | N |  |
|  |  |  |  |  | c.628G>A/c.628G>A |  |  |
| Kon, A. et al.1998 | N | N | LAMB3 | H-JEB | c.1482delC/p.W95X | N |  |
| Yasuko Takizawa et al.1998 | 3 months | N | LAMB3 | H-JEB | c.166C>T(p.Q166X)/c.2069A>G(p.W610X) | N |  |
|  | **8 months** | N |  |  |  |  |  |
| Yasuko Takizawa et al.1998 | **6 months** | F | LAMB3 | H-JEB | c.2806C>T/ c.2806C>T (p.Q936X) | N |  |
| Posteraro, P. et al.1998 | 5 years | M | LAMB3 | nH-JEB | c.904delT/c.628G>A | N |  |
| Mellerio, J. E. et al.1998 | over 30 years | M | LAMB3 | GABEB | c.628G>A(p.E210K)/c.124C>T | N |  |
|  | over 20 years | M |  |  | c.628G>A(p.E210K)/c.29insC |  |  |
|  | 2 years | M |  | JEB | c.628G>A(p.E210K)/p.R81X |  |  |
| Pulkkinen, L. et al.1998 | 30 years | F | LAMB3 | nH-JEB | c.1903C>T/c.1438del5 | N |  |
| Takizawa, Y. et al.1998 | **10 months** | M | LAMB3 | H-JEB | c.1929delCA/p.W610X | N |  |
| Cserhalmi-Friedman, P. B. et al.1998 | 42 years | M | LAMB3 | nH-JEB | c.1700ins38/c.1970G>A | N |  |
| Leena Pulkkinen et al.1997 | **2 months** | M | LAMB3 | H-JEB | c.727C>T/c.727C>T (p.Q243X) | N |  |
| Pulkkinen, L. et al.1997 | N | N | LAMB3 | H-JEB | c.1903C>T/p.R972X | N |  |
|  |  |  |  |  | c.727C>T/c.1903C>T |  |  |
|  |  |  |  |  | p.C290X/c.1903C>T |  |  |
|  |  |  |  |  | c.565-2A>G/c.1903C>T |  |  |
|  |  |  |  |  | c.1903C>T/c.2910-1G>A |  |  |
|  |  |  |  |  | c.29insC/c.1903C>T |  |  |
| Sirpa Kivirikko et al.1996 | N | N | LAMB3 | JEB | c.1903C>T (p.R635X)/ p.R144X | N |  |
|  |  |  |  |  | c.1903C>T/c.29insC |  |  |
|  |  |  |  |  | c.1903C>T/c.462insT |  |  |
|  |  |  |  |  | c.1903C>T/c.124C>T (p.R42X) |  |  |
|  |  |  |  |  | c.124C>T/c.124C>T |  |  |
|  |  |  |  |  | c.685–1G>C/c.685–1G>C |  |  |
|  |  |  |  |  | c.957ins77/p.R660X |  |  |
|  |  |  |  |  | p.R569X/c.727C>T |  |  |
| McGarth, J. A. et al.1996 | 1 year | F | LAMB3 | nH-JEB | c.1903C>T/c.628G>A | N |  |
|  | 75 years | F |  |  | c.1903C>T/c.1048A>C |  |  |
| Vailly, J. et al.1995 | **4 months** | N | LAMB3 | H-JEB | c.1760delC/c.1760delC | N |  |
| McGrath, J. A. et al.1995 | N | N | LAMB3 | GABEB | c.123C>T/c.628G>A | N |  |
| McGrath, J. A. et al.1995 | N | N | LAMB3 | H-JEB | c.957ins77/c.957ins77 | N |  |
| Pulkkinen, L. et al.1994 | N | N | LAMB3 | H-JEB | c.1903C>T/c.1903C>T | N |  |

Supplemental Table 2, M: male; F: female; N: not mentioned; JEB: Junctional Epidermolysis Bullosa; H-JEB: Herlitz Junctional Epidermolysis Bullosa; nH-JEB: non-Herlitz Junctional Epidermolysis Bullosa; JEB-GI: Junctional Epidermolysis Bullosa Generalized Intermediate; GABEB: Generalized atrophic benign epidermolysis bullosa; ages at death are in bold; mutations that have been reported before are in red.
